# Aspalathin Protects the Heart against Hyperglycemia-Induced Oxidative Damage by Up-Regulating *Nrf2* Expression

**DOI:** 10.3390/molecules22010129

**Published:** 2017-01-14

**Authors:** Phiwayinkosi V. Dludla, Christo J. F. Muller, Elizabeth Joubert, Johan Louw, M. Faadiel Essop, Kwazi B. Gabuza, Samira Ghoor, Barbara Huisamen, Rabia Johnson

**Affiliations:** 1 Biomedical Research and Innovation Platform (BRIP), Medical Research Council (MRC), Tygerberg 7505, South Africa; pdludla@mrc.ac.za (P.V.D.); christo.muller@mrc.ac.za (C.J.F.M.); johan.louw@mrc.ac.za (J.L.); kwazi.gabuza@mrc.ac.za (K.B.G.); samira.ghoor@mrc.ac.za (S.G.); bh3@sun.ac.za (B.H.); 2 Division of Medical Physiology, Faculty of Health Sciences, Stellenbosch University, Tygerberg 7505, South Africa; 3 Department of Biochemistry and Microbiology, University of Zululand, Kwadlangezwa 3886, South Africa; 4 Post-Harvest and Wine Technology Division, Agricultural Research Council (ARC) Infruitec-Nietvoorbij, Stellenbosch 7599, South Africa; JoubertL@arc.agric.za; 5 Department of Food Science, Stellenbosch University, Stellenbosch 7599, South Africa; 6 Cardio-Metabolic Research Group (CMRG), Department of Physiological Sciences, Stellenbosch University, Stellenbosch 7599, South Africa; mfessop@sun.ac.za

**Keywords:** diabetes mellitus, cardiomyopathy, hyperglycemia, oxidative stress, aspalathin, *Nrf2*

## Abstract

Aspalathin (ASP) can protect H9c2 cardiomyocytes against high glucose (HG)-induced shifts in myocardial substrate preference, oxidative stress, and apoptosis. The protective mechanism of ASP remains unknown. However, as one of possible, it is well known that phytochemical flavonoids reduce oxidative stress via nuclear factor (erythroid-derived 2)-like 2 (Nrf2) activation resulting in up-regulation of antioxidant genes and enzymes. Therefore, we hypothesized that ASP protects the myocardium against HG- and hyperglycemia-induced oxidative damage by up-regulating *Nrf2* expression in H9c2 cardiomyocytes and diabetic (*db*/*db*) mice, respectively. Using an oxidative stress RT^2^ Profiler PCR array, ASP at a dose of 1 µM was demonstrated to protect H9c2 cardiomyocytes against HG-induced oxidative stress, but silencing of *Nrf2* abolished this protective response of ASP and exacerbated cardiomyocyte apoptosis. *Db*/*db* mice and their non-diabetic (*db*/*+*) littermate controls were subsequently treated daily for six weeks with either a low (13 mg/kg) or high (130 mg/kg) ASP dose. Compared to nondiabetic mice the *db*/*db* mice presented increased cardiac remodeling and enlarged left ventricular wall that occurred concomitant to enhanced oxidative stress. Daily treatment of mice with ASP at a dose of 130 mg/kg for six weeks was more effective at reversing complications than both a low dose ASP or metformin, eliciting enhanced expression of *Nrf2* and its downstream antioxidant genes. These results indicate that ASP maintains cellular homeostasis and protects the myocardium against hyperglycemia-induced oxidative stress through activation of *Nrf2* and its downstream target genes.

## 1. Introduction

The International Diabetes Federation recently reported a rapid rise in cases of diabetes mellitus (DM) worldwide [1]. The current number of individuals living with DM is estimated to be 415 million and this figure is expected to reach 642 million by the year 2040 [1]. Diabetes is a major risk factor for the development of cardiovascular complications. Hyperglycemia, a hallmark of DM, is associated with rising cardiovascular deaths in the diabetic population [2,3]. Coronary artery disease remains the causal factor linked to the increase of cardiovascular-related deaths in diabetic persons [3]. However, diabetic cardiomyopathy (DCM) is a frequent but commonly unrecognized pathology that exists in the absence of coronary artery disease or hypertension [2,3,4,5,6]. DCM is characterized by left ventricular (LV) dysfunction leading to decreased cardiac efficiency in diabetic individuals [4,5]. Alterations in myocardial substrate preference and mitochondrial dysfunction are some of the metabolic perturbations implicated in the onset of LV dysfunction [6,7]. Oxidative stress is another factor linked with cardiac structural and functional modifications observed in a diabetic heart [8,9]. The mechanisms related to the development of oxidative injury are often multifactorial and may involve a cascade of events associated with various cell-signaling pathways [10].

Humans are equipped with a defense system that controls free radical species and reduces oxidative stress. The nuclear factor (erythroid-derived 2)-like 2 (*Nrf2*) is an emerging regulator of cellular resistance to oxidants. *Nrf2* plays a crucial role in controlling basal and induced expression of an array of cytoprotective and antioxidant defense genes important for the regulation of physiological and pathophysiological outcomes in response to oxidative exposure [11,12]. In cultured cardiomyocytes and endothelial cells, suppression of *Nrf2* expression results in oxidative stress-induced insulin resistance [13,14,15], while heart tissues isolated from *Nrf2* knockout mice display a marked increase in the production of reactive oxygen species (ROS) compared to wild type mice [13]. *Nrf2* preserves intracellular redox homeostasis by increasing the expression of antioxidant and detoxifying genes such as glutathione peroxidase 2 (*Gpx2*), superoxide dismutase (*Sod*), and NAD(P)H dehydrogenase [quinone] 1 (*Nqo1*) [16]. Under conditions of oxidative stress, *Nrf2* is activated by disassociating from its negative regulator Kelch-like ECH-associated protein 1 and translocating to the nucleus, where it binds to the antioxidant response element, activating detoxifying enzymes and genes to inactivate the stressors and restore homeostasis [17].

Various phytochemicals from food substances—such as sulforaphane, derived from a major glucoslinolate of broccoli and quercetin, an aglycone, derived from flavonol glycosides, common to plant foods, can prevent oxidative injury by up-regulating Nrf2 expression [17,18,19,20]. Several studies carried out in our laboratory demonstrated the beneficial effects of rooibos (*Aspalathus linearis*), a popular South African herbal tea and some of its major polyphenolic compounds in ameliorating metabolic complications associated with DM [21,22,23,24,25]. Aspalathin (ASP), a *C*-glucosyl dihydrochalcone unique to rooibos, can improve glucose and lipid metabolism by modulating 5′ adenosine monophosphate-activated protein kinase (AMPK) expression in 3T3-L1 adipocytes [26]. In addition to its antioxidant [27,28,29] and antidiabetic properties [30,31], in a recent study we demonstrated that ASP protects H9c2 cardiomyocytes against high glucose (HG)-induced oxidative damage by improving myocardial substrate metabolism via AMPK regulation [32]. While AMPK plays a noticeable role in regulating energy metabolism in cardiomyocytes, the control of hyperglycemia-induced oxidative stress may be additionally enhanced through other redox-related pathways such as *Nrf2* [33]. Hence, we tested the hypothesis that ASP can prevent HG- and hyperglycemia-induced oxidative injury by up-regulating the transcriptional expression of *Nrf2* by using an in vitro H9c2 cardiomyocyte model and a leptin receptor-deficient *db*/*db* mouse model, respectively.

## 2. Results

### 2.1. In Vitro Screening of ASP in H9c2 Cardiomyocytes

#### 2.1.1. ASP Maintained Cellular Homeostasis In Vitro

High glucose exposure decreased endogenous antioxidant capacity of the cells. RT^2^-PCR analysis revealed that ASP post-treatment at a dose of 1 µM was able to enhance mRNA expression of a number of antioxidant genes and phase II detoxification enzymes. Results obtained showed that ASP increased the expression of catalase (*Cat*; 11.8-fold), *Gpx2* (15.8-fold), peroxiredoxin 1, 3, 4, and 6 (2.4-, 3.0-, 2.1-, and 2.8-fold, respectively), and *Sod1* and *Sod2* (2.1- and 1.2-fold) (Table 1). The increased expression of antioxidant genes correlated with the up-regulation of glutathione (*Gsh*)-associated and reducing genes, including glutamate-cysteine ligase catalytic subunit (*Gclc*; 6.9-fold), glutamate-cysteine ligase, modifier subunit (*Gclm*; 5.8-fold) and glutathione reductase (*Gsr*; 3.2-fold) (Table 1). Increased expression of thiol reducing genes and cytoprotective genes by ASP included sulfiredoxin 1 (*Srxn1*; 6.3-fold), thioredoxin 1 (*Txn1*; 2.0-fold), thioredoxin reductase 1 (*Txnrd1*; 13.7-fold), heme oxygenase 1 (*Hmox1*; 3.9-fold), *Nqo1* (11.4-fold), and uncoupling protein 2 (*Ucp2*; 2.8-fold) (Table 1). Interestingly, the effect of ASP was not only restricted to activation of antioxidant-associated genes, but the anti-apoptotic properties of this flavonoid were also displayed as it was able to increase the expression of B-cell lymphoma 2 (*Bcl2*; 2.6-fold) and down-regulate that of Caspase 8 (*Casp8*; -1.3) (Table 1).

#### 2.1.2. ASP Regulated Expression of *Nrf2* and Its Downstream Target Genes In Vitro

**Figure 1:**
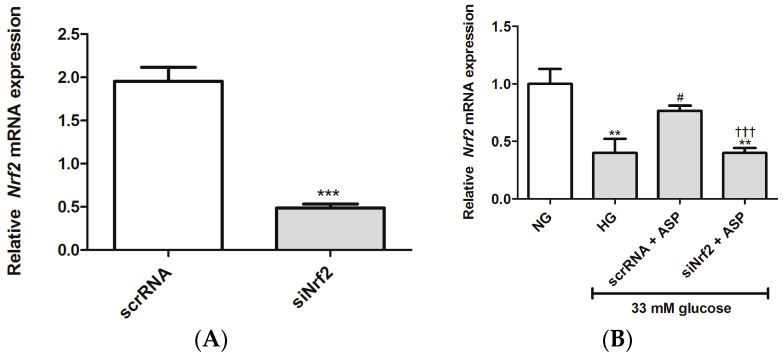
Aspalathin (ASP) increased the expression of *Nrf2* in H9c2 cardiomyocytes. (**A**) The degree of *Nrf2* knockdown in H9c2 cardiomyocytes after transfection with either small interfering RNA (siNrf2) or scrambled RNA (scrRNA) for 24 h; (**B**) H9c2 cardiomyocytes transfected with siNrf2 or scrRNA for 24 h were exposed to 33 mM glucose (HG) for 24 h, followed by treatment with 1 µM ASP for 6 h. Results are expressed as the mean ± SEM of three independent biological experiments relative to the normal glucose (NG) control, each done in triplicate. ** *p* ≤ 0.001, *** *p* ≤ 0.0001 vs. NG, ^#^ *p* ≤ 0.05 vs. HG, ^†††^ *p* ≤ 0.0001 vs. scrRNA + ASP.

To investigate if ASP activates *Nrf2*, small interfering RNA (siRNA) was employed. H9c2 cardiomyocytes exposed to siNrf2 for 24 h resulted in a significantly reduced *Nrf2* mRNA expression (0.5 ± 0.05, *p* ≤ 0.0001) when compared to the scrambled RNA (scrRNA) control (2.0 ± 0.2) (Figure 1A). Furthermore, to define the role ASP plays in the activation of *Nrf2*, HG-exposed H9c2 cells were treated with either siNrf2 or scrRNA. HG exposure decreased the expression of *Nrf2* when compared to the NG control (0.4 ± 0.12 compared to 1.0 ± 0.13, *p* ≤ 0.001) (Figure 1B). ASP treatment ameliorated this effect compared to the HG control (0.8 ± 0.04 compared to 0.4 ± 0.12, *p* ≤ 0.01) (Figure 1B). As anticipated, cells treated with siNfr2 + ASP failed to increase *Nrf2* mRNA expression when compared to the scrRNA control (Figure 1B).

**Figure 2:**
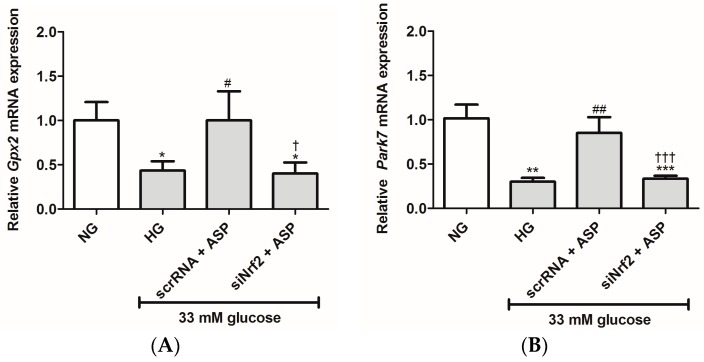

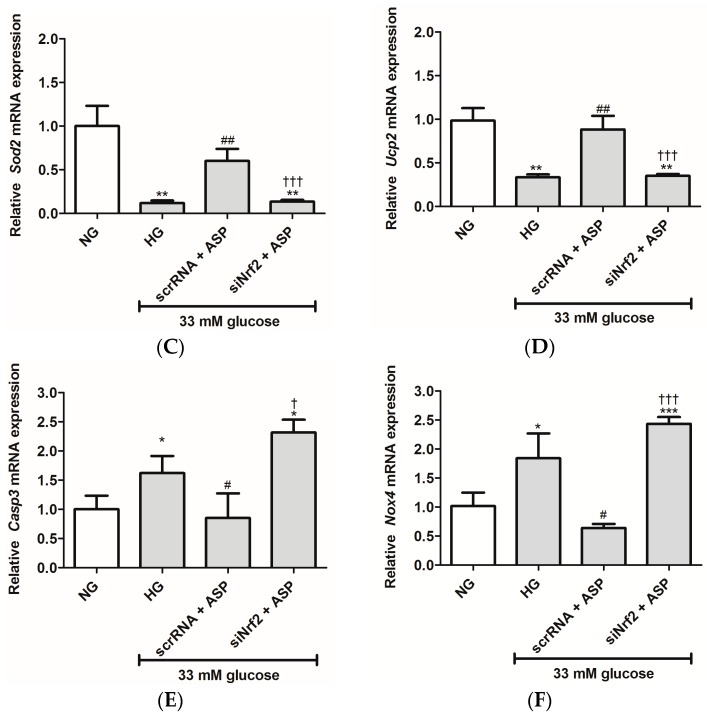
siNrf2 inhibited the effect of aspalathin (ASP) in vitro. The relative mRNA expression of antioxidant genes, including (**A**) *Gpx2*, (**B**) *Park7*, (**C**) *Sod2*, and (**D**) *Ucp2*, and oxidative damage associated genes (**E**) *Casp3* and (**F**) *Nox4* after transfection of cells with either small interfering RNA (siNrf2) or scrambled RNA (scrRNA) for 24 h followed by treatment with 1 µM ASP for 6 h. Results are expressed as the mean ± SEM of 3 independent biological experiments relative to the normal glucose (NG) control, each done in triplicate. * *p* ≤ 0.05, ** *p* ≤ 0.001, *** *p* ≤ 0.0001 vs. NG, ^#^ *p* ≤ 0.05, ^##^ *p* ≤ 0.001 vs. HG, ^†^ *p* ≤ 0.05, ^†††^ *p* ≤ 0.0001 vs. scrRNA + ASP.

The effects of siNrf2 on ASP treatment on mRNA expression of antioxidant and oxidative genes were further investigated in the study. These results show that HG exposure significantly reduced the mRNA expression of oxidative stress protective genes, *Gpx2* (0.4 ± 0.04, *p* ≤ 0.01), *Park7* (0.3 ± 0.04, *p* ≤ 0.001), *Sod2* (0.1 ± 0.03, *p* ≤ 0.001), and *Ucp2* (0.3 ± 0.33, *p* ≤ 0.001) when compared to the NG controls (1.0 ± 0.20, 1.0 ± 0.15, 1.0 ± 0.23, and 1.0 ± 0.14, respectively) (Figure 2A–D). Treatment with ASP at 1 µM for 6 h was able to prevent this by up-regulating the expression of *Gpx2* (1.0 ± 0.32, *p* ≤ 0.02), *Park7* (0.85 ± 0.17, *p* ≤ 0.001), *Sod2* (0.6 ± 0.13, *p* ≤ 0.001), and *Ucp2* (0.9 ± 0.15, *p* ≤ 0.001) when compared to the HG controls (Figure 2A–D). Interestingly, the effect on cells exposed to siNrf2 + ASP was down-regulated when compared to scrRNA + ASP and similar to when compared to HG (Figure 2A–D).

Moreover, the mRNA expression of genes associated with oxidative damage (*Casp3* and *Nox4*) was up-regulated in HG exposed cells (1.6 ± 0.43, *p* ≤ 0.04; and 1.8 ± 0.4, *p* ≤ 0.03) when compared to the NG controls (1.0 ± 0.23, and 1.0 ± 0.20, respectively) (Figure 2E,F). ASP treatment was able to abolish this effect for both *Casp3* and *Nox4* (0.9 ± 0.42, *p* ≤ 0.04; and 0.6 ± 0.06, *p* ≤ 0.01, respectively) when compared to the HG controls (Figure 2E,F). However, treatment with siNrf2 + ASP failed to reduce expression of both these genes when compared to cells subjected to scrRNA + ASP (Figure 2E,F). The effect was similar to the HG control.

### 2.2. In Vivo Confirmation Studies on db/db Mice

#### 2.2.1. Effect of ASP on Fasting Plasma Glucose (FPG) and Oral Glucose Tolerance Tests (OGTTs)

**Figure 3:**
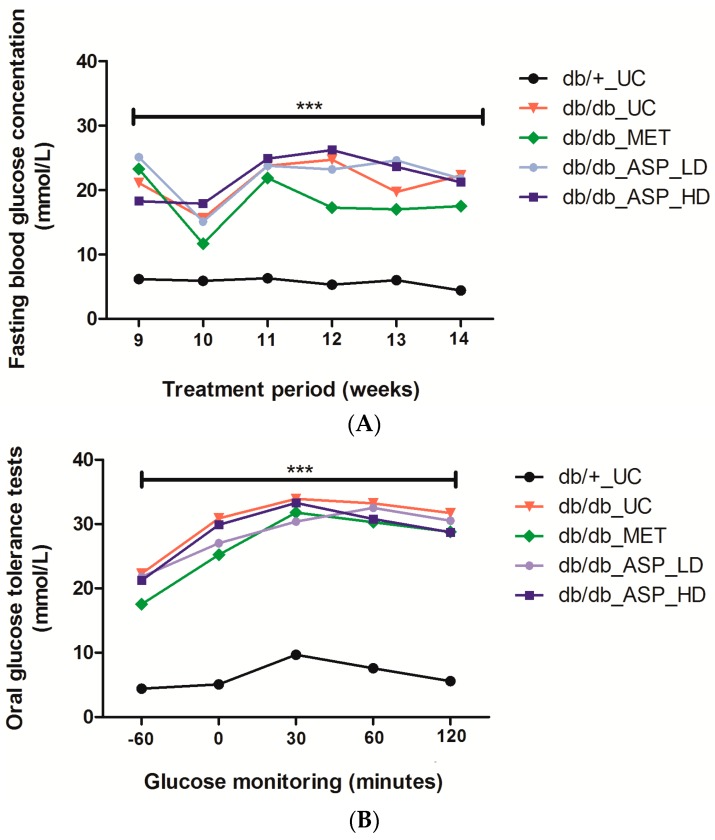
Aspalathin (ASP) treatment did not reduce increasing fasting plasma glucose (FPG) levels, however a high dose of 130 mg/kg of ASP was able to improve oral glucose tolerance in diabetic mice after 60 and 120 minutes from baseline. (**A**) FPG levels (**B**) Oral glucose tolerance tests (−60: baseline reading). Results are expressed as the mean ± SEM and each treatment group contained six mice. *** *p* ≤ 0.0001 diabetic control mice (*db*/*db*_UC) vs. untreated nondiabetic mice (*db*/*+*_UC). *db*/*db*_MET: diabetic mice treated with metformin (150 mg/kg); *db*/*db*_ASP_LD: diabetic mice treated with aspalathin low dose (13 mg/kg); *db*/*db*_ASP_HD: diabetic mice treated with aspalathin high dose (130 mg/kg).

Male C57BLKS/J homozygous (*db*/*db*) mice and their heterozygous leptin-receptor-deficient nondiabetic lean littermate controls (*db*/*+*) were treated daily with either a low (13 mg/kg) or a high (130 mg/kg) dose of ASP, as well as metformin (MET) at 150 mg/kg for six weeks. The untreated diabetic control (*db*/*db*_UC) group displayed elevated FPG levels from week 9 to week 14 (21.0 ± 1.73, 15.5 ± 1.29, 23.7 ± 1.15, 24.7 ± 2.91, 19.7 ± 1.46, and 22.3 ± 2.85, respectively) when compared to the untreated *db*/*+*_UC control group (6.1 ± 0.30, 5.9 ± 0.50, 6.2 ± 0.38, 5.3 ± 0.44, 6.0 ± 0.20, and 4.3 ± 0.49, respectively) (Figure 3A). Although both the low (13 mg/kg) and high dose (130 mg/kg) ASP treatments did not reduce the increased FPG levels in *db*/*db* mice, MET was able to reduce the raised FPG levels from week 12 to week 14 (17.3 ± 1.92, 17.0 ± 1.67, and 17.4 ± 2.56, respectively), though not significantly (Figure 3A).

#### 2.2.2. Assessment of FPG Levels after Administration of a 2 g/kg Glucose Bolus in Mice

Results obtained showed a marked increase in FPG levels 30 min after glucose administration, and this was significantly different when the *db*/*db*_UC (33.8 ± 0.40, *p* ≤ 0.0001) was compared to the *db*/*+*_UC control (9.6 ± 0.50) (Figure 3B). High dose ASP treatment at 60 and 120 min (30.8 ± 1.53 and 28.6 ± 1.63) was comparable to MET (30.2 ± 1.61 and 28.8 ± 2.22) in reducing the increased FPG concentrations when compared to the untreated diabetic controls, though not significantly (Figure 3B). Treatment with a low dose of ASP was, however, ineffective.

#### 2.2.3. ASP Prevented Hyperglycemia-Induced LV Mass Enlargement In Vivo

**Figure 4:**
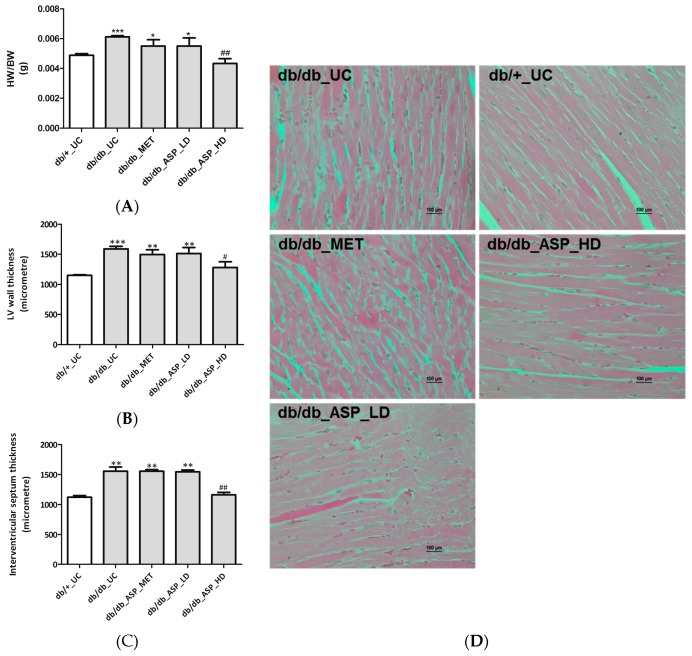
Aspalathin (ASP) prevented hyperglycemia-induced cardiac muscle structure modifications associated with hypertrophy in diabetic mice. (**A**) Heart weight (HW) to body weight (BW) ratio; (**B**) left ventricular (LV) wall thickness; (**C**) interventricular septum thickness; and (**D**) photomicrographs of cardiac remodelling. Results are expressed as the mean ± SEM and each treatment group contained six mice. * *p* ≤ 0.05, ** *p* ≤ 0.001, *** *p* ≤ 0.0001 vs. untreated nondiabetic mice (*db*/*+*_UC), ^#^ *p* ≤ 0.05, ^##^ *p* < 0.001 vs. untreated diabetic mice (*db*/*db*_UC). *db*/*db*_MET: diabetic mice treated with metformin (150 mg/kg); *db*/*db*_ASP_LD: diabetic mice treated with aspalathin low dose (13 mg/kg); *db*/*db*_ASP_HD: diabetic mice treated with aspalathin high dose (130 mg/kg).

Heart hypertrophy associated measurements, including heart weight (HW)/body weight (BW) ratio, LV wall and interventricular septum thickness, and occurrence of cardiac muscle remodeling were assessed after mice were treated with ASP for six weeks. Results showed that the *db*/*db*_UC group presented with increased HW/BW ratio (0.0061 ± 0.00005, *p* ≤ 0.0001), LV wall and interventricular septum thickness measurements (1588 ± 42, *p* ≤ 0.0001 and 1556 ± 70, *p* ≤ 0.001, respectively) occurring concurrent to enhanced cardiac remodeling in comparison to the *db*/*+*_UC control group (HW/BW ratio: 0.0048 ± 0.0001, LV wall: 1151 ± 9; and interventricular septum: 1120 ± 29) (Figure 4A–D). Interestingly, treatment with a high dose ASP was able to ameliorate these dysregulations associated with cardiac hypertrophy, including prevention of changes in HW/BW ratio (0.0043 ± 0.0003, *p* ≤ 0.001), LV wall (1278 ± 96, *p* = 0.01), and interventricular septum thickness (1160 ± 42, *p* ≤ 0.001) (Figure 4A–D). Neither MET nor a low dose ASP had any effect on the altered cardiac muscle fibers or ventricular wall thickness.

#### 2.2.4. ASP Regulated the Expression of *Nrf2* and Its Target Genes In Vivo

**Figure 5:**
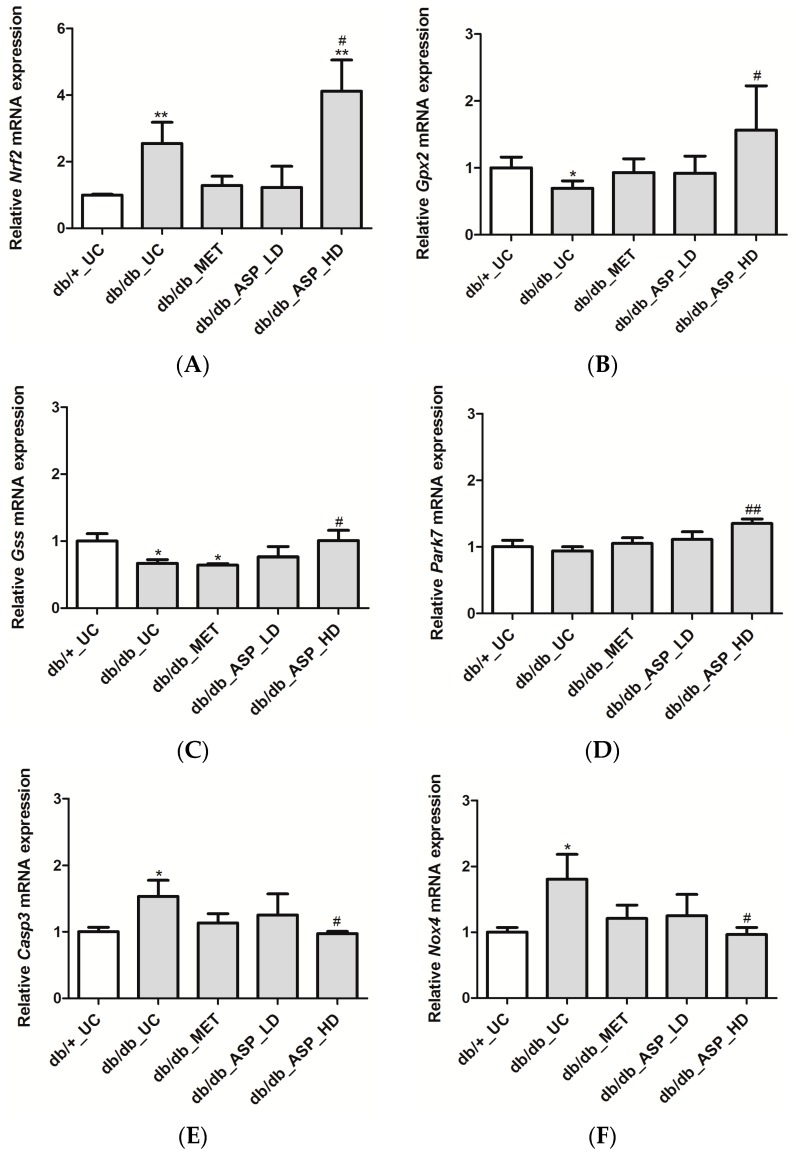
Aspalathin (ASP) prevented oxidative damage by increasing the expression of *Nrf2* and its down-stream target genes in diabetic mice. The relative mRNA expression of (**A**) *Nrf2* and associated antioxidant genes, including (**B**) *Gpx2*, (**C**) *Gss*, and (**D**) *Park7*, and oxidative damage associated genes (**E**) *Casp3* and (**F**) *Nox4* after the six-week treatment period with metformin, and a low or high dose ASP. Results are expressed as the mean ± SEM and each treatment group contained six mice. * *p* ≤ 0.05, ** *p* ≤ 0.001 vs. untreated nondiabetic mice (*db*/*+*_UC), ^#^ *p* ≤ 0.05, ^##^ *p* ≤ 0.001 vs. untreated diabetic mice (*db*/*db*_UC). *db*/*db*_MET: diabetic mice treated with metformin (150 mg/kg); *db*/*db*_ASP_LD: diabetic mice treated with aspalathin low dose (13 mg/kg); *db*/*db*_ASP_HD: diabetic mice treated with aspalathin high dose (130 mg/kg).

In support of the in vitro data on H9c2 cardiomyocytes, we assessed the expression of *Nrf2* and its associated downstream cytoprotective genes in vivo, and results showed a significant enhancement of *Nrf2* mRNA expression in the *db*/*db*_UC group (2.54 ± 0.63, *p* ≤ 0.001) in comparison to the *db*/*+*_UC control group (0.99 ± 1.49) (Figure 5A). Although MET and low dose ASP treatments were not effective, the high dose ASP (4.1 ± 0.93, *p* ≤ 0.01) significantly increased mRNA expression of *Nrf2* when compared to both *db*/*db*_UC and *db*/*+*_UC groups (Figure 5A). 

Further analyzing mRNA expression of genes activated by *Nrf2*, it was noted that the expression of *Gpx2* (0.69 ± 0.11, *p* ≤ 0.04) and *Gss* (0.66 ± 0.05, *p* ≤ 0.03) was reduced in the *db*/*db*_UC group when compared to the *db*/*+*_UC group (1.0 ± 0.15, 1.0 ± 0.11, respectively) (Figure 5B,C). The expression of *Park7* was not significantly affected (0.94 ± 0.06). Treatment with MET and the low dose ASP did not have any effect, while the high dose ASP significantly up-regulated expression of *Gpx2* (1.56 ± 0.66, *p* ≤ 0.04), *Gss* (1.0 ± 0.15, *p* ≤ 0.03), and *Park7* (1.35 ± 0.06, *p* ≤ 0.001) when compared to *db*/*db*_UC group (Figure 5B–D).

Expression of oxidative damage-associated genes (*Casp3* and *Nox4*) was significantly enhanced in the *db*/*db*_UC group (1.53 ± 0.24, *p* ≤ 0.02; and 1.8 ± 0.37, *p* ≤ 0.01) when compared to the *db*/*+*_UC group (1.0 ± 0.06; and 1.0 ± 0.07, respectively) (Figure 5E,F). no effect was observed for met or low dose asp treatments, while the high dose asp treatment was able to down-regulate the expression of both *Casp3* (0.97 ± 0.03, *p* ≤ 0.01) and *Nox4* (0.96 ± 0.10, *p* ≤ 0.01) when compared to *db*/*db*_UC group (Figure 5E,F).

## 3. Discussion

Accumulative evidence has shown that certain dietary phytochemicals are able to activate *Nrf2* and thereby increase its cytoprotective response [12,16,18]. Presence of a catechol group may be a pivotal structural feature of flavonoids that contributes to *Nrf2* activation as demonstrated for shogaol derivatives [34]. Furthermore, a dihydrochalcone glycoside, neohesperidin dihydrochalcone, has recently been shown to prevent carbon tetrachloride-induced hepatic injury by increasing the expression of *Hmox1* and *Nqo1* through activation of *Nrf2* in HepG2 cells [35]. In agreement with others [18,19,35], our study showed that activation of *Nrf2* prevented oxidative stress by increasing the expression of an array of antioxidant genes and enzymes. We showed that a 6 h treatment with 1 µM ASP protected cardiomyocytes exposed to HG against oxidative damage by up-regulating the expression of antioxidant genes and cytoprotective enzymes such as *Nqo1* and *Hmox1*. This was consistent with a dramatic increase in the expression levels of *Cat*, *Txnrd1*, *Gpx2*, and *Gsh*-synthesizing subunits (*Gclc* and *Gclm*). This is an essential result since reduced expression of these antioxidant genes is consistently reported in a diabetic heart tissue [23,36]. Txnrd is crucial in reducing the oxidised form of Txn, whereas Cat and Gpx2 are important in the detoxification of hydrogen peroxide. Increased regulation of *Gpx2*, which is mainly expressed in the gastrointestinal tract and liver was not anticipated due to its limited expression in the heart [37,38]; however, additional studies are required to validate these findings, especially focusing on protein expression levels.

Moreover, by using RNA interference we showed that activation of *Nrf2* by ASP is crucial for the antioxidant response against HG-induced oxidative injury in H9c2 cardiomyocytes. While it was expected that *Nrf2*-knockdown may exacerbate susceptibility of such cells to oxidative damage, ASP treatment showed an even higher capacity to enhance *Nrf2* expression and its downstream target genes and thus protect against HG-induced stress. However, an isolated in vitro cell-based system is not influenced by any variables, thus the observed decrease in *Nrf2* after HG exposure of H9c2 cells. Whereas, in an in vivo system the expression and regulation of genes is influenced by various factors. In this study, we speculate that in the *db*/*db* mice, the observed increase of *Nrf2*-expression in the *db*/*db*_UC group was a compensatory mechanism used by the cells to decrease increased oxidative stress.

Therefore, following in vitro experiments on H9c2 cardiomyocytes, it was important to confirm such findings in an in vivo system. For this we made use of *db*/*db* mice, which represent a type 2 diabetic mouse model. These mice display a whole-body phenotype of DM and are frequently used in the pharmacological study of compounds including ASP [30,39]. Moreover, they have been reported to spontaneously become obese and develop hyperglycemia from the age of eight weeks [40]. At an age of 10–18 weeks, these mice display signs of cardiac interstitial fibrosis and hypertrophy as a result of increased LV wall thickness, occurring simultaneous to enhanced generation of ROS [40,41].

Accelerated ROS production induces deleterious modifications to DNA, proteins and lipids concomitant to the pathophysiological state observed within the diabetic heart [42,43]. To support this, we showed that, in addition to impaired OGTT, *db*/*db* mice presented elevated FPG levels, an increased HW/BW ratio that occurred concomitant to a thickened LV wall and cardiac remodelling when compared to the *db*/*+* control group. An enlarged heart LV wall is a conspicuous sign of DCM and is identified as an early defect associated with chronic hyperglycemia in diabetic patients [4,6]. The latter state has been associated with elevated oxidative stress and various studies have reported on the effect of oxidative stress on vascular remodeling and how antioxidant therapy prevents progressive remodeling and improves cardiac function [6,8,18,32]. Correspondingly, in this study we observed that morphological derangements within *db*/*db* mouse hearts occurred concurrently to accelerated oxidative stress and a modest increase in *Nrf2* expression. It has been previously reported that the expression of *Nrf2* is initially elevated in response to stress, and then diminishes at a later stage leading to adverse complications such as apoptosis [18,44].

Despite the limited protective effect displayed by ASP treatment in reducing elevated FPG concentrations, this polyphenol at a dose of 130 mg/kg was able to ameliorate oxidative damage via *Nrf2* activation, resulting in reduced LV wall thickness and cardiac remodelling. This was an important result since most polyphenols have been shown to induce pro-oxidant effect at higher concentrations [23,45]. However, an unexpected finding was the inability of ASP to attenuate increased FPG concentrations, since it has been previously reported to have blood glucose lowering effects [21,30,31]. A similar finding was observed where treatment of streptozotocin-induced diabetic mice with sulforaphane failed to reduce FPG, but was able to protect the heart against oxidative stress associated with heart tissue remodelling [18]. The inability of ASP to reduce blood glucose can also be due to other factors, such as the duration of treatment, as our group has previously demonstrated that ASP reduces blood glucose concentrations in streptozotocin-induced diabetic rats over a period of 6 h [21]. In a study done by Kawano et al. [30], the authors showed that ASP was able to suppress increased FPG levels of *db*/*db* mice for five weeks as well as improve impaired glucose tolerance at 30, 60, 90, and 120 min. However, based on our model to induce cardiomyopathy at 9–14 weeks, the disease had to be at an advance stage with chronically elevated blood glucose levels and thus we did not expect ASP or MET to have an effect on hyperglycemia. Similarly, another factor that could influence the analysis of results, especially the confidence intervals and *p*-values, could be the small sample size as this has been reported previously [46,47].

In contrast, while current antidiabetic agents including MET effectively reduce FPG concentrations, there is still an increasing number of cardiovascular-related deaths in diabetic patients [3]. Correspondingly, this study showed that even though MET-treated mice presented with moderately reduced FPG levels, it was unable to prevent oxidative stress associated myocardial structural modifications. This result supports current evidence reporting on the increasing number of natural products and medicinal plants being tested in combination with current drugs such as MET for the prevention and treatment of type DM [48,49,50]. An interesting finding from our group has shown that ASP has no inhibitory effect on cytochrome P450 enzymes [50], suggesting that it could be used in combination with glucose lowering agents such as thiazolidinediones or sulfonylureas to alleviate oxidative injury within a diabetic heart. However, to date, no pharmacokinetics study has been performed on ASP, but it will form part of our groups future investigations. Therefore, additional studies where *db*/*db* mice are treated with a combination of ASP and MET before measuring relevant markers associated with hyperglycemia-induced myocardial injury are of foremost importance.

In summary, consistent with previous findings, ASP displays strong properties to improve myocardial ultrastructure by preventing HG-associated complications. However, dose selection remains important to induce desired efficacy, since the low dose of 13 mg/kg did not show any effect in protecting the heart when compared to the high dose of 130 mg/kg in our study. In addition to its modulatory effects of AMPK, the ability of ASP to regulate *Nrf2* may be a key factor to protect against DCM. However, further studies to assess the impact of ASP on cardiac functional parameters in diabetic rodents are required to confirm this exciting proposal. In addition, an in-depth study is being planned looking at *Nrf2*, mitochondrial function, and ASP protection, with the use of more robust molecular techniques.

## 4. Materials and Methods

### 4.1. Reagents and Kits

H9c2 rat derived cardiomyoblasts (ECACC No. 8809294) were purchased from the European Collection of Cell Cultures (Salisbury, Wiltshire, UK), while ASP was synthesized according to Han et al. [51] and obtained from High Force Research (ca. 98%, batch SZI-356-54) (Durham, UK). Hematoxylin, eosin, xylene, and formalin were obtained from Merck-Millipore (Billerica, MA, USA), halothane was from Safeline Pharmaceuticals (Johannesburg, South Africa), Dulbecco’s Modified Eagle’s Medium, Dulbecco’s phosphate-buffered saline, penicillin, and streptomycin from Lonza (Verviers, Belgium), and fetal bovine serum and horse serum from Biochrom (Berlin, Germany). High Capacity Reverse Transcription Kit, RNAse free water, siNrf2 (cat #AM16708), scrRNA (cat #AM4615), TRIzol reagent, Turbo DNase Kit, Lipofectamine RNAimax reagent, and all Taqman gene expression assays were supplied by ThermoFisher Scientific, Inc. (Waltham, MA, USA). Rat Oxidative Stress and Atherosclerosis RT^2^ Profiler PCR Arrays (PARN-065ZA and PARN-065ZA, respectively), RT^2^ SYBR Green qPCR Master Mix, RT^2^ Array First Strand Kit and RNeasy Mini Kit were obtained from Qiagen (Valencia, CA, USA). All other consumables and reagents were purchased from Sigma-Aldrich Corp. (St. Louis, MO, USA), unless otherwise specified.

### 4.2. In Vitro Experiments on H9c2 Cardiomyocytes

#### 4.2.1. Cell Culture

H9c2 cardiomyoblasts were cultured in supplemented Dulbecco’s Modified Eagle’s Medium (10% fetal bovine serum, 100 μg/mL penicillin and 100 μg/mL streptomycin) overnight under standard tissue culture conditions (37 °C in humidified air and 5% CO_2_). Cells were seeded in a 6-well plate at a seeding density of 2 × 10^4^ cells/well. Confluent H9c2 cardiomyoblasts were differentiated into adult cardiomyocytes by substituting growth media with differentiation media consisting of Dulbecco’s Modified Eagle’s Medium supplemented with 10 nM all-trans-retinoic acid and 1% horse serum for six days [52]. On day seven, differentiated cells were exposed to 33 mM glucose for 48 h prior to treatment with ASP (1 µM) for an additional 6 h. All treatment doses of HG and ASP were based on a previous study [32]. Cells exposed to either 5.5 mM glucose or 33 mM glucose served as controls for normal glucose (NG) and HG, respectively. Cells exposed to 33 mM mannitol were used to rule out the effect of osmolarity [32].

#### 4.2.2. Preparation of ASP for Cell Culture Treatment

A stock solution of ASP (22.1 mM), prepared in dimethyl sulfoxide, was diluted using Dulbecco’s Modified Eagle’s Medium to give a final solution of 1 µM. Concentrations of ASP have been previously optimized [32]. The toxic effect of dimethyl sulfoxide at a concentration of 0.004% was tested and ruled out in all the experiments performed. In addition, the effect of ASP on NG treated cells has been previously investigated and shows no toxicity [53].

#### 4.2.3. RNA Isolation and Purification

Total RNA was extracted using Trizol reagent, according to a previously described protocol [32]. RNA was purified using an RNeasy Mini Kit, while the Turbo DNase Kit was used to remove genomic DNA, as per manufacturer’s instructions. RNA integrity was determined using an Agilent 2100 Bioanalyser (Agilent Technologies, Inc., Palo Alto, CA, USA), according to manufacturer’s instructions.

#### 4.2.4. RT^2^-PCR Array Analysis

cDNA was synthesized from 2 µg RNA using the RT^2^ First Strand Kit, according to manufacturer’s instructions. Rat Oxidative Stress and Atherosclerosis RT^2^ Profiler PCR Arrays were used (PARN-065ZA and PARN-065ZA) to analyse the differential expression of multiple genes. Briefly, cDNA was added to the RT^2^ SYBR Green qPCR Master Mix and aliquoted onto each well of the 96-well RT^2^ Profiler PCR Array plate. An ABI 7500 Instrument (ThermoFisher Scientific, Inc., USA) was used for mRNA quantification at the following cycling conditions: 50 °C for 1 min, 1 cycle of 95 °C for 10 min, followed by 40 cycles of 95 °C for 15 s and 60 °C for 1 min. Analysis of PCR array data was done using a Microsoft Excel sheet with macros made available by the manufacturer (http://pcrdataanalysis.sabiosciences.com/pcr/arrayanalysis.php). Each array contained five housekeeping genes (*Actb*, *B2m*, *Gapdh*, *Gusb*, and *Hsp90ab1*) against which the sample data were normalized. The transcript level of each candidate gene was quantified according to the ΔΔ*C*t method. *C*t values > 35 were not included in the analysis and considered as negative.

#### 4.2.5. Real-Time PCR to Confirm Oxidative Stress and Apoptosis Markers

Quantitative RT-PCR was performed on an ABI 7500 Instrument. cDNA was synthesized from 1 µg of RNA using the High Capacity Reverse Transcription Kit, as per manufacturer’s instructions. Thereafter, the PCR reaction mix was prepared by adding 5 µL Taqman universal PCR master mix, 0.5 µL Taqman gene expression assay (in vitro study (*Casp3*, *Gpx2*, *Nox4*, *Nrf2*, *Park 7*, *Sod2*, and *Ucp2*) and in vivo study (*Casp3*, *Gpx2*, *Gss*, *Nox4*, *Nrf2*, and *Park 7*)), 1 µL of cDNA, and 3.5 µL RNAse free water to a final volume of 10 µL. The quantitative RT-PCR protocol was conducted as follows: 50 °C for 1 min and 95 °C for 10 min, followed by 40 cycles of 95 °C for 15 s and 60 °C for 30 s. Gene expression data were normalized to hypoxanthine-guanine phosphoribosyltransferase (*Hprt*). Of note, not all genes quantified in the RT^2^ Array plate were repeated for the siRNA study, but only those that have been previously shown to be affected by ASP [32].

#### 4.2.6. Knockdown of Nrf2 Using Small Interfering RNA

Nrf2-siRNA was done using a Lipofectamine RNAimax reagent, according to the manufacturer′s instructions. Briefly, H9c2 cells at approximately 70% confluence were transfected with siNrf2 or scrRNA for 24 h, respectively. Thereafter, transfected cells were exposed to HG for 24 h before treatment with ASP for 6 h. The degree of Nrf2 knockdown after 24 h of transfection was confirmed by RT-PCR. Non-transfected cells, exposed to either 5.5 mM glucose or 33 mM glucose served as controls for NG and HG, respectively.

### 4.3. In Vivo Experiments Using C57BL/KS Mice

#### 4.3.1. Animals

*Db/db* and *db/+* were obtained from Jackson’s Laboratories (Sacramento, CA, USA) and housed at the Primate Unit and Delft Animal Centre (PUDAC) of the South African Medical Research Council (SAMRC) in a controlled environment with a 12 h light/dark cycle in a temperature range of 23–25 °C (relative humidity: ~50%). The mice received standard laboratory chow pellets (Afresh Vention, Cape Town, South Africa) ad libitum and had free access to water. Ethical clearance for the use of animals in this study was granted by the SAMRC Ethics Committee for Research on Animals (ECRA No. 07/13), and the Stellenbosch University Ethics Committee (SU-ACUM13-00021).

#### 4.3.2. Treatment of Mice with ASP

Nine-week old diabetic (*db*/*db*) mice and their non-diabetic (*db*/*+*) littermate controls were treated daily for six weeks through oral gavage with either a low (13 mg/kg) or high (130 mg/kg) ASP dose and compared to MET at a dose of 150 mg/kg. Dose selection for ASP and MET was based on previous studies [32,33]. Treatment groups (*n* = 6/group) included; (i) *db*/*+* untreated controls (*db*/*+*_UC); (ii) *db*/*db* untreated controls (*db*/*db*_UC); (iii) *db*/*db*_MET (*db*/*db*_MET); (iv) *db*/*db* ASP low dose (*db*/*db*_ASP_LD); (v) and *db*/*db* ASP high dose (*db*/*db*_ASP_HD). ASP and MET were dissolved in distilled water before orally administration at the same time (08:00–09:00) every day for six weeks, while untreated animals were given water in place of treatment.

#### 4.3.3. Heart Tissue Staining and Left Ventricular Hypertrophic Measurements

After the six-week treatment period, mice were fasted for 4 h before being weighed and anesthetized with halothane. Animals received the anesthetic until no reaction could be recorded by pedal reflex before removal of the heart. The heart tissue was weighed and fixed in 10% formalin for a minimum of 16 h before it was processed using a Leica TP 1020 automated processor (Leica Biosystems, Buffalo Grove, IL, USA) and embedded in paraffin wax. Paraffin embedded tissue was cut into sections and attached to aminopropyltriethoxysilane coated glass slides. Tissues were then stained with H&E as previously described [54]. Stained sections were visualized using a Nikon Eclipse Ti inverted microscope (Tokyo, Japan). Micrographs for heart LV and median wall thickness were taken in non-overlapping fields of 1 mm^2^ under 40× magnification and measurements were done using NIS Elements imaging software (Tokyo, Japan).

#### 4.3.4. Measurement of FPG Concentrations

In mice fasted overnight for 16 h, FPG concentrations were measured by tail prick using a OneTouch Select handheld glucometer (LifeScan Inc., Milpitas, CA, USA), according to the manufacturer’s instructions.

#### 4.3.5. OGTTs

After the six weeks of treatment, OGTTs were performed. Briefly, after a 16 h fast, a glucose bolus of 2 g/kg was orally administered through gastric gavage. Plasma glucose levels were determined by tail prick at time intervals of 0, 30, 60, and 120 min.

### 4.4. Statistical Analysis

Data were expressed as the mean ± SEM. Results for in vitro experiments were expressed as the mean of three independent biological experiments with each experiment containing at least three technical replicates, unless otherwise stated. For in vivo experiments, each treatment group contained six mice. Statistical analysis was performed using GraphPad Prism software version 5.00 (GraphPad Software, Inc., La Jolla, CA, USA). Comparisons between groups were performed using one-way multivariate ANOVA, followed by unpaired Student *t*-test, and a *p*-value of ≤ 0.05 was deemed as statistically significant.

## Figures and Tables

**Table 1:** Aspalathin treatment (1 µM) increased the expression of antioxidant genes and phase II cytoprotective enzymes in H9c2 cardiomyocytes pre-exposed to 33 mM glucose for 48 h.

Gene	Fold Regulation after High Glucose Exposure	Fold Regulation After Post-Treatment with Aspalathin
**Antioxidant genes**		
Catalase (*Cat*)	−1.63	11.80
Glutathione peroxidase 2 (*Gpx2*)	−1.20	15.86
Peroxiredoxin 1 (*Prdx1*)	−1.33	2.49
Peroxiredoxin 3 (*Prdx3*)	−1.21	3.09
Peroxiredoxin 4 (*Prdx4*)	−1.72	2.14
Peroxiredoxin 6 (*Prdx6*)	−2.65	2.89
Superoxide dismutase 1 (*Sod1*)	−1.25	2.18
Superoxide dismutase 2 (*Sod2*)	−1.17	1.22
**Glutathione synthesis genes**		
Glutamate-cysteine ligase catalytic subunit (*Gclc*)	−1.84	6.96
Glutamate-cysteine ligase, modifier subunit (*Gclm*)	−1.40	5.89
Glutathione reductase (*Gsr*)	−1.50	3.29
**Reducing agent genes**		
Sulfiredoxin 1 (*Srxn1*)	−3.43	6.34
Thioredoxin 1 (*Txn1*)	−1.08	2.05
Thioredoxin reductase 1 (*Txnrd1*)	−2.88	13.70
Thioredoxin reductase 2 (*Txnrd2*)	−3.25	1.08
**Cytoprotective genes**		
Heme oxygenase 1 (*Hmox1*)	−2.77	3.98
NAD(P)H dehydrogenase (quinone 1) (*Nqo1*)	−4.57	11.45
Uncoupling protein 2 (*UCP2*)	−3.95	2.83
Uncoupling protein 3 (*UCP3*)	−2.01	−1.61
**Apoptotic genes**		
B-cell lymphoma 2 (*Bcl2*)	−1.8	2.6
Caspase 8 (*Casp8*)	3.9	−1.3
